# Membrane-Associated Ubiquitin Ligase RING Finger Protein 152 Orchestrates Melanogenesis via Tyrosinase Ubiquitination

**DOI:** 10.3390/membranes14020043

**Published:** 2024-02-01

**Authors:** Ryota Ueda, Rina Hashimoto, Yuki Fujii, José C. J. M. D. S. Menezes, Hirotaka Takahashi, Hiroyuki Takeda, Tatsuya Sawasaki, Tomonori Motokawa, Kenzo Tokunaga, Hideaki Fujita

**Affiliations:** 1 Faculty of Pharmaceutical Sciences, Nagasaki International University, Sasebo 859-3298, Japan; uedar@niu.ac.jp (R.U.); yfujii@niu.ac.jp (Y.F.);; 2 Esteem Industries Pvt Ltd., Bicholim 403529, Goa, India; 3 Proteo-Science Center, Ehime University, Matsuyama 790-8577, Japan; takahashi.hirotaka.rn@ehime-u.ac.jp (H.T.); takeda.hiroyuki.mk@ehime-u.ac.jp (H.T.); sawasaki.tatsuya.mf@ehime-u.ac.jp (T.S.); 4 Frontier Research Center, POLA Chemical Industries, Inc., Yokohama 244-0812, Japan; motchymotchy@gmail.com; 5 Department of Pathology, National Institute of Infectious Diseases, Tokyo 162-8640, Japan

**Keywords:** lysosome, melanogenesis, melanosome, RNF152, tyrosinase, ubiquitin ligase

## Abstract

Lysosomal degradation of tyrosinase, a pivotal enzyme in melanin synthesis, negatively impacts melanogenesis in melanocytes. Nevertheless, the precise molecular mechanisms by which lysosomes target tyrosinase have remained elusive. Here, we identify RING (Really Interesting New Gene) finger protein 152 (RNF152) as a membrane-associated ubiquitin ligase specifically targeting tyrosinase for the first time, utilizing AlphaScreen technology. We observed that modulating RNF152 levels in B16 cells, either via overexpression or siRNA knockdown, resulted in decreased or increased levels of both tyrosinase and melanin, respectively. Notably, RNF152 and tyrosinase co-localized at the trans-Golgi network (TGN). However, upon treatment with lysosomal inhibitors, both proteins appeared in the lysosomes, indicating that tyrosinase undergoes RNF152-mediated lysosomal degradation. Through ubiquitination assays, we found the indispensable roles of both the RING and transmembrane (TM) domains of RNF152 in facilitating tyrosinase ubiquitination. In summary, our findings underscore RNF152 as a tyrosinase-specific ubiquitin ligase essential for regulating melanogenesis in melanocytes.

## 1. Introduction

The abundance of membrane proteins directly correlates with their activity and physiological functions in eukaryotic cells. For instance, the functionality of ion channels and receptors is partially dependent on their abundance and correct cellular localization [1]. Ubiquitination of membrane proteins serves as a targeting signal for either proteasomes or lysosomes, contingent upon the site of occurrence [2,3]. When ubiquitination of membrane proteins occurs in the endoplasmic reticulum (ER), these proteins are extracted from the ER membrane to the cytosol, where they undergo proteasomal degradation, which is known as ER-associated degradation (ERAD) [4]. Conversely, when ubiquitination of membrane proteins occurs within post-Golgi compartments, such as the plasma membrane, trans-Golgi network (TGN), or endosomes, these ubiquitinated proteins are directed toward late endosomes (LEs)/multivesicular bodies (MVBs). Subsequently, they are sorted into intraluminal vesicles of MVBs where they undergo degradation through MVBs-lysosome fusion [5,6]. During these steps, a multitude of ubiquitin ligases participate in the ubiquitin-dependent control of endocytosis and lysosomal degradation of membrane proteins [7].

Numerous soluble cytosolic ubiquitin ligases (e.g., Nedd4-1, WWP1, WWP2, SMURF1, SMURF2, and Cbl) directly or indirectly recognize their specific substrate membrane proteins and facilitate their ubiquitination. Membrane-associated ubiquitin ligases, including most of the MARCH family and some members of the RNF family, are localized in specific compartments of the secretory/endocytic system, where their substrate membrane proteins are also localized. MARCH1, -2, -3, and -8 are localized in endosomes and/or lysosomes and ubiquitinate their respective substrates [8,9,10,11].

RNF152, a membrane-associated RING (Really Interesting New Gene)-finger ubiquitin ligase, is localized in lysosomes and reportedly plays a role in lysosome-related apoptosis [12]. RNF152 also acts as a negative regulator of the mTORC1 pathway by targeting RagA for K63-linked ubiquitination [13]. RNF152 acts as a positive regulator of TLR/IL-1R-mediated signaling by promoting oligomerization of MyD88 [14]. Additionally, RNF152 targets TSPAN12 for ubiquitination and subsequent proteasomal degradation, resulting in the suppression of hepatocellular carcinoma cell proliferation [15]. RNF152, along with RNF182, RNF183, and RNF186, constitutes the RNF183 family, consisting of closely related genes encoding a RING-finger domain (C3HC4) at their N-terminus and one or two predicted transmembrane domains at their C-terminus with high homology [16].

The abundance of tyrosinase, a key enzyme in melanin synthesis, directly affects melanogenesis (cell pigmentation level). Tyrosinase is translated in the ER as a type I membrane glycoprotein, trafficked via the Golgi, and delivered to melanosomes, where melanin is synthesized and accumulated [17,18,19,20,21,22]. Tyrosinase is known to undergo various post-translational modifications, including glycosylation, sequential trimming, phosphorylation, and copper loading during its transport through secretory pathways [23]. Moreover, *S-palmitoylation* of the cytoplasmic tail of tyrosinase accelerates its degradation, leading to the inhibition of melanogenesis [24]. Certain whitening reagents reduce the protein level of tyrosinase without altering its transcription level [25], often resulting in the degradation of tyrosinase through proteasomal or lysosomal activity. Linoleic acid induces proteasomal degradation of tyrosinase, presumably via the ERAD pathway [26,27], while phenylthiourea (PTU) [28] and inulavosin [29,30] induce lysosomal degradation of tyrosinase. Recently PTU has been reported to exhibit an inhibitory effect on cell pigmentation in zebrafish by triggering the activation of autophagy [31]. In the past decades, several studies have highlighted novel reagents that decrease tyrosinase levels, resulting in hypopigmentation [32,33,34,35,36]. However, despite the effects of these reagents, the mechanisms responsible for selectively directing tyrosinase toward ERAD and lysosomal degradation pathways remain unclear. Elucidating the processes through which cells or whitening reagents regulate melanogenesis will necessitate the identification and characterization of tyrosinase-specific ubiquitin ligases. In this study, employing AlphaScreen technology, we identified RNF152 as a membrane-associated ubiquitin ligase that specifically targets tyrosinase for ubiquitination. Our findings reveal that this ubiquitin ligase directs tyrosinase toward lysosomal degradation, ultimately regulating melanogenesis.

## 2. Experimental Procedures

### 2.1. Antibodies and Reagents

Rabbit polyclonal antibodies against mouse tyrosinase and tyrosinase-related protein-1 (Tyrp-1) were described previously [29]. Rabbit polyclonal antibody against synthetic peptide of mouse RNF152 (G_145_APPEA VEEEPDRRGV VK_162_) was obtained from Sigma-Aldrich (St. Louis, MO, USA) and evaluated (Supplementary Materials, Figure S1). The mouse monoclonal antibody against human LAMP1 (1D4B) was generously provided by Dr. K. Furuta (National Cancer Center Research Institute, Tokyo, Japan). Mouse monoclonal antibodies against β-actin and c-myc and rabbit antibodies against HA and c-myc were purchased from Sigma-Aldrich. The goat antibody against HA was from GenScript (Piscataway, NJ, USA). The mouse anti-ubiquitin monoclonal antibody (FK2) was from Nippon Bio-Test Laboratories (Tokyo, Japan). All secondary antibodies for immunofluorescence analysis were purchased from Molecular Probes (Eugene, OR, USA). The protease inhibitor cocktail was from Nacalai Tesque (Kyoto, Japan). *N*-ethylmaleimide was from Sigma-Aldrich. Pepstatin A, leupeptin, and E64d were from the Peptide Institute (Osaka, Japan). Bafilomycin A1 was from BioViotica (San Diego, CA, USA). Protein A-coupled Sepharose 4B was from GE Healthcare UK (Buckinghamshire, UK).

### 2.2. Cell Culture

Mouse melanoma B16, HEK293T, and HeLa cells were obtained from JCRB Cell Bank (Osaka, Japan). Mouse melanoma B16 cells were cultured in MEM supplemented with 10% FBS. HEK293T and HeLa cells were cultured in DMEM supplemented with 10% FBS. All cells were maintained at 37 °C in a humidified 5% CO_2_ atmosphere.

### 2.3. Screening for E3 Ubiquitin Ligases That Interact with Tyrosinase

A protein array containing 224 biotinylated human and mouse E3 ligases was constructed using a wheat germ cell-free synthesis system [37]. V5 epitope-tagged tyrosinase and dihydrofolate reductase (DHFR) were synthesized with this system. The binding of V5-tagged tyrosinase to the protein array was assayed using AlphaScreen technology with slight modification from previous protocols [38]. In summary, 1 μL of the cell-free synthesized V5-tagged bait protein and 1 μL of biotinylated E3 protein were mixed in a 15 μL AlphaScreen buffer containing 100 mM of Tris-HCl (pH 8.0), 0.01% of Tween 20, 100 mM of NaCl, and 1 mg/mL of BSA in an OptiPlate 384 titer plate (PerkinElmer; Waltham, MA, USA). This protein mixture was incubated for 1 h at 26 °C, followed by the addition of a 10 μL detection mixture (0.01 µL of anti-V5 antibody (Invitrogen; Carlsbad, CA, USA), 0.1 μL of streptavidin-conjugated AlphaScreen donor beads, and 0.1 μL of protein A-conjugated AlphaScreen acceptor beads in AlphaScreen buffer), and incubated for another 1 h at 26 °C. AlphaScreen luminescent signal was measured using an EnVision multilabel plate reader (PerkinElmer), and the relative luminescent signal was calculated by dividing the value obtained from the interaction between the E3 ligase and tyrosinase by that obtained from the E3 ligase and DHFR interaction.

### 2.4. Plasmid Construction and Transfection, siRNA and Transfection

The cDNAs encoding human RNF152 or mouse tyrosinase and mouse Tyrp-1 were PCR-amplified and subcloned into either pcDNA3.1/Myc-His(-) B (Invitrogen) or pCMV-HA-C (Clontech; Palo Alto, CA, USA). The primer sets used were as follows: RNF152 (5′- ATA GAATTC ATG GAG ACG CTG TCC CAG GAC TCT CTG C-3′, 5′-ATA GGATCC GCC ACA GGA TAT CAC AGT GAA GCG CTT AG-3′); mouse tyrosinase (5′- ATA GAATTC ATG TTC TTG GCT GTT TTG TAT TGC CTT CTG TGG-3′, 5′-ATA GTCGAC G CAG ATG GCT CTG ATA CAG CAA GCT GTG GTA GTC G-3′); and mouse Tyrp-1 (5′-ATA GAATTC ATG AAA TCT TAC AAC GTC CTC CCC CTA GCC-3′, 5′-ATA GTCGAC G GAC CAT GGA GTG GTT AGG ATT CGG GAG CTC-3′). Two mutant RNF152-myc plasmids were obtained from GenScript (Tokyo, Japan). The 3×FLAG-ubiquitin plasmid was described previously [9]. siRNAs, ON-TARGETplus Mouse RNF152 siRNA, and ON-TARGETplus Non-targeting Control siRNAs were purchased from Horizon Discovery (Tokyo, Japan). For transfection in B16 cells, TransFast (Roche Molecular Biochemicals; Indianapolis, IN, USA) was used as per the manufacturer’s instructions. For transfection in HeLa cells, FuGENE 6 (Roche Molecular Biochemicals) was employed following the manufacturer’s instructions. For B16 cell siRNA transfection, RNAiMAX (Thermo Fisher Scientific; Waltham, MA, USA) was used as per the manufacturer’s instructions.

### 2.5. Melanin Content Measurement

The melanin content was measured using the method described by Kim et al. [39] with slight modification. B16 cells (3 × 10^5^ cells) were seeded on a 60 mm dish and subsequently transfected with either the plasmids (RNF152-myc or empty vector) or siRNAs (ON-TARGETplus Mouse RNF152 or ON-TARGETplus Non-targeting Control siRNAs). The transfected cells were directly solubilized with 1 M NaOH and boiled at 80 °C for 30 min. The dissolved melanin content was then measured using spectrophotometry at OD_405_. The values were normalized to the protein concentration and presented as percentages relative to the control. It should be noted that seeding more than 3 × 10^5^ cells was required to obtain consistent results, thereby increasing the signal-to-noise ratio for this assay.

### 2.6. Immunoprecipitation (IP) and Western Blotting

Total cell lysates and immunoprecipitated proteins were prepared as described previously [9]. Briefly, cells were lysed in TBS-T buffer (50 mM Tris-HCl buffer (pH 7.5), 0.15 M NaCl, 1% Triton X-100, and 0.5% deoxycholic acid) containing a protease inhibitor cocktail and 10 mM of *N*-ethylmaleimide. The lysate was centrifuged at 21,500× *g* for 15 min, and the supernatant was used as the total cell lysate for immunoblotting or IP. Protein A-coupled Sepharose 4B was pre-incubated with appropriate antibodies for 2 h at 4 °C. The total cell lysate was then incubated with antibody-coupled Sepharose overnight at 4 °C and washed three times with TBS-T buffer. Immunoprecipitated proteins were eluted with SDS sample buffer and subjected to SDS-PAGE, and then the immunoreactive bands were visualized using an ECL detection kit (GE Healthcare). The protein concentration of the total cell lysates was measured using a BCA assay kit (Thermo Fisher Scientific) and an equal amount of proteins were loaded for cell lysate immunoblotting. The bands were scanned with the ChemiDoc system (Bio-Rad, Japan), and band intensities were quantitated with Image J software version 1.47t (NIH, MD, USA). The band intensities of proteins of interest were normalized with β-actin.

### 2.7. Confocal Immunofluorescence Microscopy

B16 and HeLa cells were cultured on coverslips, fixed with 4% paraformaldehyde for 30 min on ice, and permeabilized with 0.05% saponin (Sigma-Aldrich). Fixed cells were subjected to immunofluorescence analysis as described previously [9]. In brief, fixed coverslips were stained with primary antibodies: mouse anti-myc monoclonal antibody (Sigma-Aldrich) and either rabbit anti-tyrosinase or anti-Tyrp-1 antibody. For double staining, secondary antibodies, Alexa 488 donkey anti-mouse IgG (A-21202) and Alexa 568 donkey anti-rabbit IgG (A-10042), were used. Fixed coverslips were also stained with rabbit anti-myc antibody (Sigma-Aldrich), goat anti-HA antibody (GenScript), and either mouse anti-syntaxin 6 (BD Biosciences; London, UK) or anti-LAMP1 antibody. For triple staining, the secondary antibodies used were Alexa 488 donkey anti-goat IgG (A-11055), Alexa 568 donkey anti-rabbit IgG (A-10422), and Alexa 647 donkey anti-mouse IgG (A-31571). Confocal images were captured using FluoView FV10i (Olympus; Tokyo, Japan) or STELLARIS 5 (Leica Microsystems, Tokyo, Japan). The Pearson’s correlation coefficient was calculated as a statistical measure to evaluate co-localization. This analysis was conducted using FIJI software in Image J plugin Coloc 2. version 2.14.0/1.54h, incorporating region of interest (ROI) data from confocal images.

### 2.8. Statistical Analyses

Either column graphs or box-and-whisker plots, with individual data points, were created with GraphPad Prism version 10.1.2. All values are presented as the mean ± s.d. for three independent experiments. Statistical comparisons were made using an unpaired *t*-test or one-way analysis of variance and Dunn’s multiple comparisons test and a *p* < 0.05 was considered statistically significant.

## 3. Results

### 3.1. Tyrosinase Ubiquitination in B16 Melanoma Cells

**Figure 1:**
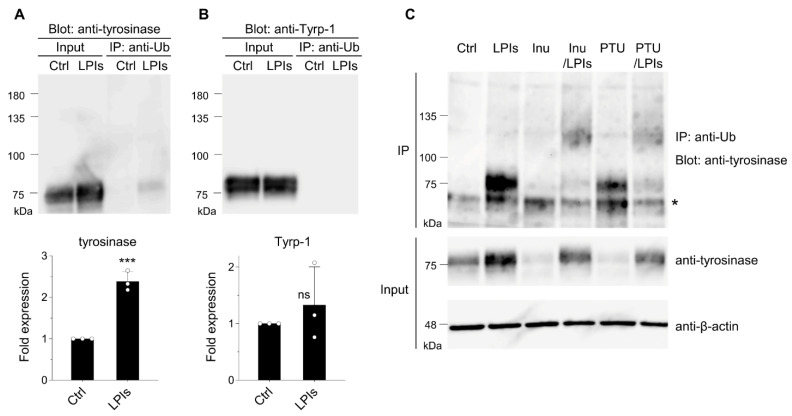
Tyrosinase undergoes ubiquitination and degradation in lysosomes in B16 melanoma cells. (**A**,**B**) Cells were treated with either DMSO (control, Ctrl) or LPIs (leupeptin, pepstatin A, E64d; each 40 μM) for 24 h. Equal amounts of cell lysates, quantified using a commercial assay kit, were subjected to immunoblotting with either an anti-tyrosinase or anti-Tyrp-1 antibody. Additionally, lysates were immunoprecipitated with an anti-ubiquitin antibody (IP: anti-Ub) followed by immunoblotting using the anti-tyrosinase or anti-Tyrp-1 antibody. The expression levels of tyrosinase were quantified in the bands and presented as fold expression relative to the Ctrl (mean ± s.d., *n* = 3). *** *p* ≤ 0.001 compared with the Ctrl using two-tailed unpaired *t*-tests. ns, not significant. (**C**) Cells were treated with DMSO (control, Ctrl), LPIs, inulavosin (Inu; 15 μM), inulavosin combined with LP (Inu/LP), PTU (PTU; 100 μM), or PTU combined with LP (PTU/LP) for 24 h. Lysates were immunoprecipitated with the anti-ubiquitin antibody followed by immunoblotting with the anti-tyrosinase antibody (“IP: anti-Ub Blot: anti-tyrosinase”), or directly immunoblotted with the anti-tyrosinase or an anti-β-actin antibody. An asterisk indicates the presumed position of the mouse immunoglobulin G heavy chain.

Detection of protein ubiquitination involves IP of total ubiquitinated proteins using an anti-ubiquitin antibody, combined with immunoblotting using an antibody against the protein of interest [40]. To assess tyrosinase ubiquitination in B16 melanoma cells, we employed an anti-ubiquitin antibody along with an anti-tyrosinase antibody. Analysis of cell lysates revealed a robust increase in tyrosinase protein levels in the presence of lysosomal protease inhibitors (LPIs), whereas the levels of Tyrp-1, another melanosome membrane protein, remained unchanged (input shown in Figure 1A,B, with corresponding quantitative data presented below the blots). This disparity suggests varying turnover rates between these proteins [29]. We observed smear bands representing ubiquitinated tyrosinase along with the normal-sized (76-kDa) tyrosinase in total ubiquitinated proteins when LPIs were present (Figure 1A, IP). As tyrosinase forms homo-oligomers [41], this suggests ubiquitination might occur on a portion of the oligomer subunit. Corresponding experiments using anti-Tyrp-1 antibody did not display smear bands or normal-sized Tyrp-1.

The impact of inulavosin and PTU on tyrosinase ubiquitination in B16 cells was investigated. Both inulavosin [29] and PTU [28] accelerate the tyrosinase degradation rate in lysosomes, and decreased tyrosinase protein levels in our experiments (Figure 1C, anti-tyrosinase in Input, lanes Inu, and PTU). However, this degradation was restored in the presence of LPIs (Figure 1C, anti-tyrosinase in Input, lanes Inu/LP, and PTU/LPIs). Substantially increased levels of recovered ubiquitinated tyrosinase (smear bands with higher molecular weights) were observed in the presence of inulavosin or PTU combined with LPIs (Figure 1B, lanes Inu/LPIs, and PTU/LPIs in IP).

### 3.2. Identification of Tyrosinase-Specific E3 Ubiquitin Ligase

A screening process was conducted to identify ubiquitin ligases specific to tyrosinase. Using an E3 ubiquitin ligase protein array, we synthesized V5-tagged tyrosinase and DHFR (as negative control) and employed AlphaScreen technology to detect the binding of V5-tyrosinase to the protein array (see Section 2.3 “Screening for E3 ubiquitin ligases that interact with tyrosinase”). We previously demonstrated that inulavosin interferes directly with copper loading into the luminal domain of tyrosinase, leading to the formation of apo-tyrosinase, and proposed that apo-tyrosinase, possessing certain conformational defects, is selectively delivered to lysosomes [30]. In this study, we performed screenings both in the absence and presence of inulavosin. We assumed that if inulavosin induces a conformational change of tyrosinase, it would likely enhance its binding to the ubiquitin ligase in our assay. Out of 67 candidate ligases displaying a relative luminescent signal > 15 in the absence of inulavosin, 44 showed higher affinity to V5-tyrosinase in the presence of inulavosin (Table S1), and the top five candidates are shown in Table 1. Among these, RNF152 exhibited the highest affinity for tyrosinase, leading to its selection for subsequent analyses.

### 3.3. RNF152 Regulates Expression of Tyrosinase in B16 Cells

**Figure 2:**
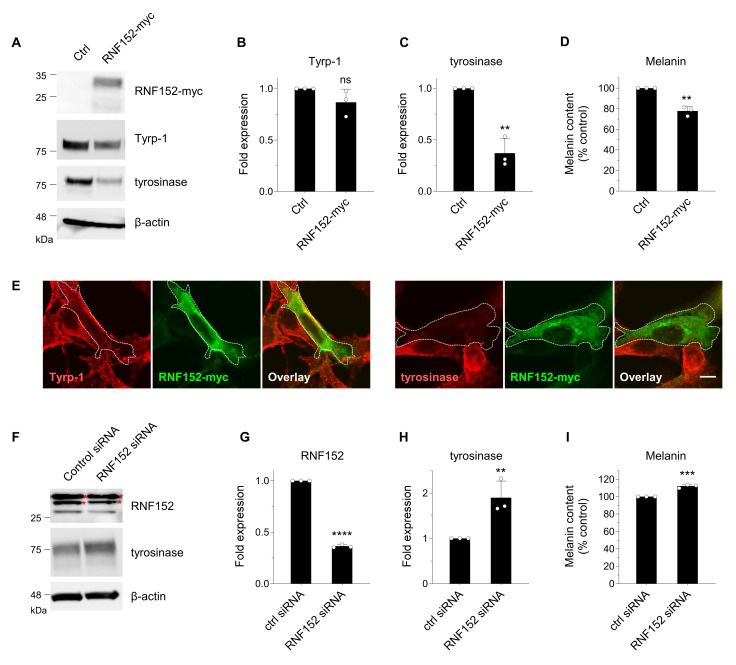
RNF152 modulation influences tyrosinase expression in B16 cells. (**A**) Cells were transfected with either an empty vector (Ctrl) or RNF152-myc. Lysates were immunoblotted with anti-tyrosinase, anti-Tyrp-1, anti-β-actin, and anti-myc antibodies. (**B**,**C**) The expression levels of Tyrp-1 (**B**) and tyrosinase (**C**) were quantified in the bands and presented as fold expression relative to the Ctrl (mean ± s.d., *n* = 3). ** *p* ≤ 0.01 compared with the Ctrl using two-tailed unpaired *t*-tests. ns, not significant. (**D**) Cells were transfected with either an empty vector (Ctrl) or RNF152-myc, and melanin content was measured using spectrophotometry. The data are shown as the percentage relative to the Ctrl (mean ± s.d., *n* = 3). ** *p* ≤ 0.01 compared with the Ctrl using two-tailed unpaired *t*-tests. (**E**) Cells transfected with RNF152-myc were subjected to immunofluorescence analysis with an anti-myc antibody and either an anti-tyrosinase or anti-Tyrp-1 antibody. Dotted white lines indicate transfected cells. Scale bar: 10 μm. (**F**) Cells were transfected with either control or RNF152 siRNA. Lysates were immunoblotted with anti-RNF152, anti-tyrosinase, or anti-β-actin antibody. Red asterisks indicate non-specific bands in the RNF152 blotting (see Figure S1). (**G**,**H**) The expression levels of RNF152 (**G**) and tyrosinase (**H**) were quantified in the bands and presented as fold expression relative to the Ctrl (mean ± s.d., *n* = 3). ** *p* ≤ 0.01, **** *p* < 0.0001 compared with the Ctrl using two-tailed unpaired *t*-tests. (**I**) The melanin content data are shown as the percentage relative to the Ctrl (mean ± s.d., *n* = 3). *** *p* ≤ 0.001 compared with the Ctrl using two-tailed unpaired *t*-tests.

We next examined the effects of RNF152 expression levels on tyrosinase and melanin content in B16 cells. Cells transfected with myc-tagged mouse RNF152 (RNF152-myc) demonstrated significantly reduced levels of tyrosinase and melanin, but not of Tyrp-1 (Figure 2A–D). It should be noted that exogenously expressed RNF152-myc displayed doublet bands when probed with the anti-myc antibody, for reasons currently unknown. However, these findings align with similar results reported previously [12]. Immunofluorescence analysis of transfected cells exhibited a specific reduction in tyrosinase but not in Tyrp-1 (Figure 2E). Inversely, transfecting siRNA that targets mouse RNF152 significantly reduced the expression of endogenous RNF152, accompanied by simultaneous increases in both tyrosinase expression and melanin content (Figure 2F–I). These results suggest that both endogenously and exogenously expressed RNF152 lead to the degradation of tyrosinase, thereby reducing melanin content in B16 melanoma cells.

### 3.4. RNF152 Co-Localizes with Tyrosinase in TGN and Degrades It in Lysosomes

**Figure 3:**
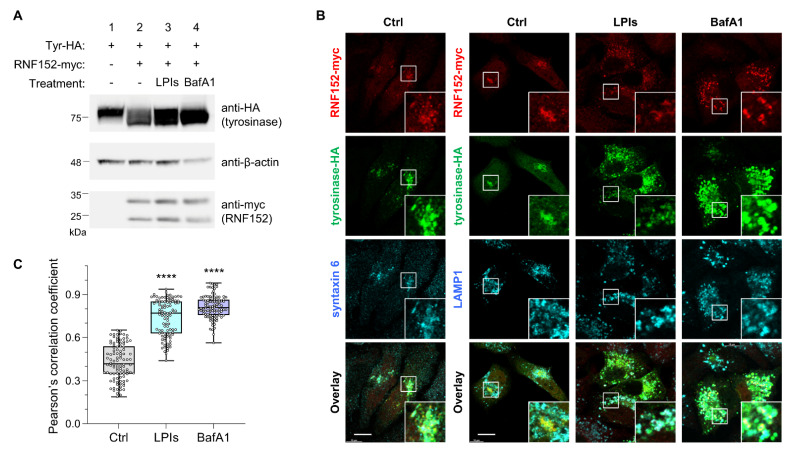
RNF152 is co-localized with tyrosinase-HA in the TGN and degrades it in lysosomes. (**A**) HEK293T cells transfected with tyrosinase-HA (Tyr-HA) alone (lane 1), or RNF152-myc and Tyr-HA in combination (lanes 2, 3, and 4), were treated with DMSO (control; lanes 1 and 2), LPIs (leupeptin, pepstatin A, E64d; each 40 μM; lane 3), or bafilomycin A1 (BafA1, 5 μM; lane 4) for 14 h. Lysates were immunoblotted with anti-HA, anti-β-actin, and anti-myc antibodies. (**B**) HeLa cells transfected with RNF152-myc and Tyr-HA were treated with DMSO (Ctrl), LPIs, or bafilomycin A1 (BafA1) as in (A), fixed, and subjected to immunofluorescence analysis with anti-myc and anti-HA antibodies, together with either an anti-syntaxin 6 (TGN marker), or anti-LAMP1 (LEs/lysosome marker) antibody. Squares indicate magnified regions. Scale bar: 10 μm. (**C**) Quantitative analyses were performed using immunofluorescence data obtained in the experiments depicted in (B). The Pearson’s correlation coefficient between tyrosinase and LAMP1 in RNF152-myc-positive cells treated with DMSO (Ctrl), LPIs, or BafA1 is shown (*n* = 100 for each treatment). Data are presented in box-and-whisker plots with the minimum, maximum, sample median, and first versus third quartiles. **** *p* < 0.0001, compared with Ctrl using one-way analysis of variance and Dunnett’s multiple-comparison tests.

To assess the routes of RNF152-induced tyrosinase degradation, HEK293T cells were transfected with HA-tagged mouse tyrosinase (tyrosinase-HA) alone or together with both RNF152-myc and tyrosinase-HA. These cells were treated with bafilomycin A1 (vacuolar H^+^-ATPase inhibitor) or LPIs for 14 h, followed by immunoblotting analysis (Figure 3A). RNF152-myc prompted the degradation of tyrosinase-HA (Figure 3A, anti-HA, compare lane 1 with lane 2), but this degradation was restored in the presence of LPIs and bafilomycin A1 (Figure 3A, anti-HA, compare lane 2 with lanes 3 and 4). Next, to evaluate the intracellular localization and degradation pathways of tyrosinase and RNF152, HeLa cells transfected with tyrosinase-HA and RNF152-myc were subjected to immunofluorescence analysis following treatment with LPIs and bafilomycin A1 (Figure 3B). Exogenously expressed tyrosinase-HA along with RNF152-myc were localized at a perinuclear region and demonstrated co-localization with syntaxin 6 (TGN marker), while exhibiting no co-localization with LAMP1 (LEs and lysosome marker) (Figure 3B, control (DMSO)). In contrast, in the presence of LPIs or bafilomycin A1, their expression levels were elevated, and they were observed within enlarged vesicular structures, demonstrating co-localization with LAMP1 (Figure 3B, BafA1 and LPIs). It should be noted that treatments with LPIs and BafA1 inherently induce morphological alterations in lysosomes, including their enlargement, likely attributed to the accumulation of undegraded proteins within these organelles [42,43]. To quantify co-localization, we calculated Pearson’s correlation coefficient between tyrosinase and LAMP1 in RNF152-myc-positive cells (Figure 3C). The results showed a significant increase in the co-localization index in the cells treated with LPIs or BafA1, compared with those treated with DMSO (Ctrl).

### 3.5. RNF152 Physically Associates with Tyrosinase

**Figure 4:**
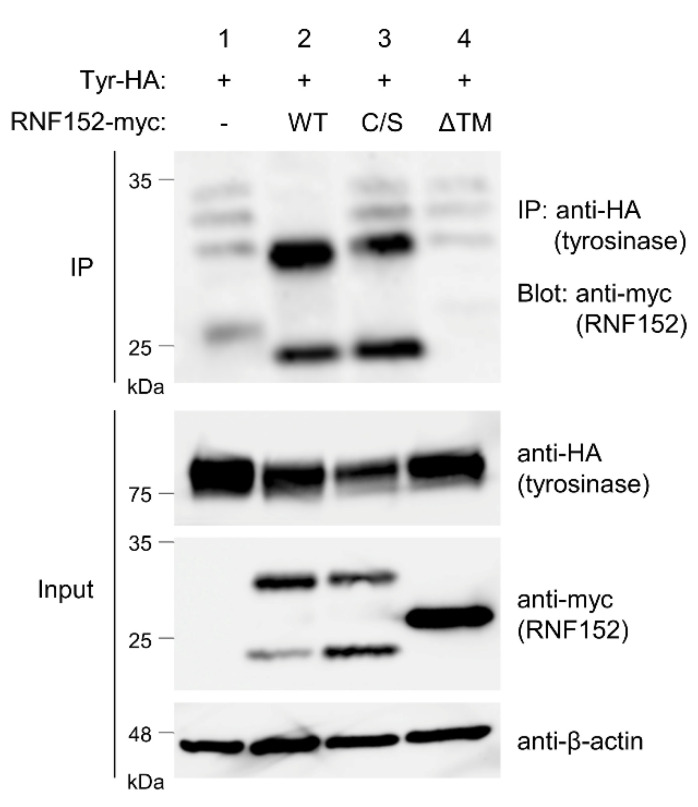
Interaction of RNF152 with tyrosinase. Lysates of HEK293T cells transfected with tyrosinase-HA (Tyr-HA) with an empty vector (lane 1), WT RNF152-myc (lane 2), C/S mutant RNF152-myc (lane 3), or ΔTM mutant (lane 4) were immunoblotted with anti-HA, anti-myc, or anti-β-actin antibody. Lysates were also immunoprecipitated with the anti-HA antibody, and immune complexes were immunoblotted with anti-myc antibody.

RNF152 comprises a luminal domain, a single transmembrane (TM) domain, and a cytoplasmic region, including a RING domain responsible for ubiquitination activity. To evaluate the binding ability and ubiquitination activity of RNF152 toward tyrosinase, we generated plasmids encoding mutant forms of RNF152: RNF152(C/S) (in which four cysteine residues located at the RING domain were replaced by serine) and RNF152(ΔTM) (in which the TM domain located at the C-terminus of RNF152 was deleted). The interaction between RNF152 and tyrosinase was assessed via co-IP analysis in HEK293T cells cotransfected with plasmids encoding tyrosinase-HA and either wild-type (WT) or mutant RNF152-myc. Cell lysate blotting revealed two bands for WT and the C/S mutant, and a single band for the ΔTM mutant (Figure 4, anti-myc in Input) consistent with that reported in [12]. Co-IP analysis revealed that both WT and C/S mutants were co-immunoprecipitated with tyrosinase-HA, whereas the ΔTM mutant displayed no tyrosinase binding ability (Figure 4, IP). Whereas the C/S mutant localized to the TGN, the ΔTM mutant was observed in the cytosol in immunofluorescence analyses, as expected (Figure S2). These differences in subcellular localization likely correspond to their respective binding capacities to tyrosinase.

### 3.6. RNF152 Strongly Ubiquitinates Tyrosinase

**Figure 5:**
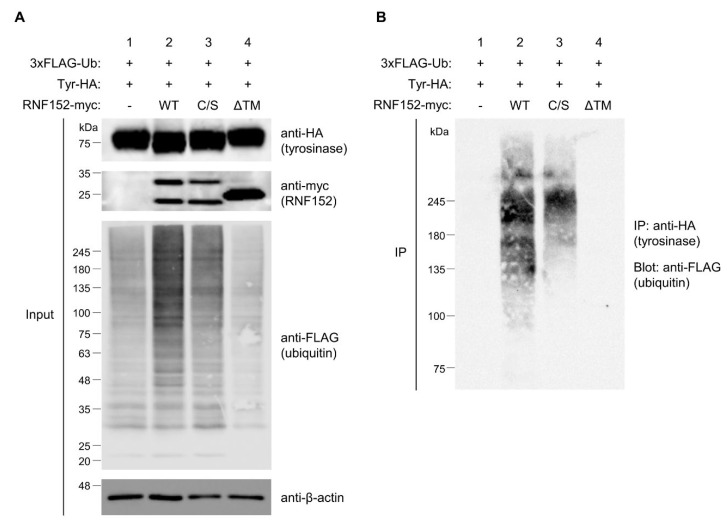
Tyrosinase ubiquitination by RNF152. (**A**) Lysates from HEK293T cells transfected with 3×FLAG-ubiquitin (Ub) and tyrosinase-HA (Tyr-HA) with empty vector (lane 1), WT RNF152-myc (2), C/S mutant (lane 3), or ΔTM mutant (lane 4) were immunoblotted with anti-HA, anti-myc, anti-β-actin, and anti-FLAG antibodies. (**B**) Lysates were immunoprecipitated with the anti-HA antibody, and immune complexes were immunoblotted with the anti-FLAG antibody. It should be noted that, for the ubiquitin assay using 3×FLAG-ubiquitin, LPIs were added to the cell culture medium for the last 12 h to enhance the signal of ubiquitination.

To assess the roles of the RING and TM domains of RNF152 in tyrosinase ubiquitination, we conducted a high-sensitivity ubiquitin assay using a 3×FLAG-ubiquitin plasmid in HEK293T cells. Cotransfection of tyrosinase-HA and 3×FLAG-ubiquitin with either WT or mutant RNF152-myc was performed. Blotting of total cell lysate with anti-FLAG antibody revealed a significant enhancement in the ubiquitination state of any substrate proteins for WT RNF152, as evidenced by smear bands or ladder bands (Figure 5A, anti-FLAG, compare lane 1 with lane 2). In contrast, their ubiquitination state was marginally reduced in the presence of the C/S mutant and remained unaffected by the ΔTM mutant (Figure 5A, anti-FLAG, compare lane 2 with lanes 3 and 4). Immunoprecipitation of tyrosinase-HA from the cell lysate using an anti-HA antibody, followed by immunoblotting with an anti-FLAG antibody revealed notably enhanced smear bands, signifying strongly ubiquitinated tyrosinase for WT RNF152, whereas smear bands were faint for the C/S mutant and absent for the ΔTM mutant (Figure 5B, compare lane 2 with lanes 3 and 4). Note that differences in their respective subcellular localization (Figure S2) likely reflect variations in their ubiquitination activity.

### 3.7. RNF152 Ubiquitinates Tyrp-1 to a Lesser Degree

**Figure 6:**
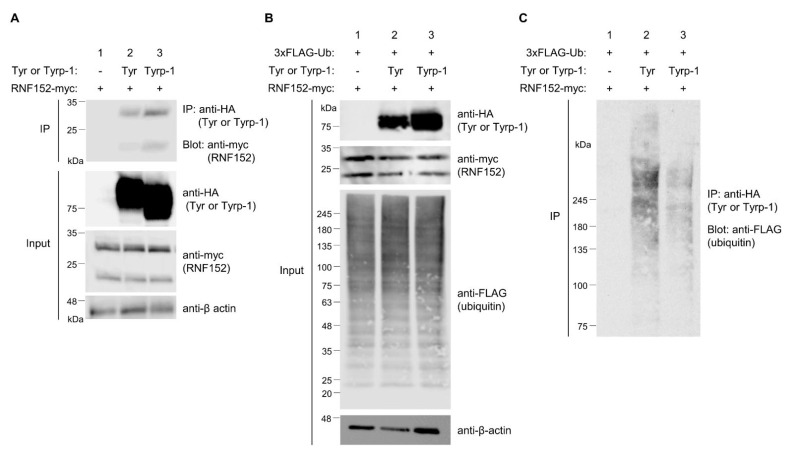
Ubiquitination of tyrosinase and Tyrp-1 by RNF152. (**A**) Lysates from HEK293T cells transfected with RNF152-myc with an empty vector (lane 1), tyrosinase-HA (Tyr-HA) (lane 2), or Tyrp-1-HA (lane 3) were immunoblotted with anti-HA, anti-myc, and anti-β-actin antibodies. Lysates were also immunoprecipitated with the anti-HA antibody, and immune complexes were immunoblotted with the anti-myc antibody. (**B**) Lysates from HEK293T cells transfected with 3×FLAG-ubiquitin and RNF152-myc with an empty vector (lane 1), Tyr-HA (lane 2), or Tyrp-1-HA (lane 3) were immunoblotted with anti-HA, anti-myc, anti-β-actin, or anti-FLAG antibody. (**C**) Lysates were processed as in Figure 5B. Note that LPIs were added to the culture medium for the purpose described in the legend of Figure 5.

Initial observations in B16 cells did not reveal Tyrp-1 ubiquitination (Figure 1A). We further explored the potential interaction of exogenously expressed RNF152-myc and its ubiquitination of Tyrp-1-HA (Figure 6A–C). Co-IP analysis showed that RNF152-myc interacted with Tyrp-1-HA to a similar extent as with tyrosinase-HA (Figure 6A, compare lane 2 with lane 3 in IP). Using a high-sensitivity ubiquitination assay with 3×FLAG-ubiquitin, we observed that RNF152-myc enhanced Tyrp-1-HA ubiquitination, albeit to a lesser degree than tyrosinase-HA ubiquitination (Figure 6C, compare lane 2 with lanes 3).

## 4. Discussion

Lysosomal degradation and ubiquitination of tyrosinase have been described in numerous studies [26,27,28,29,30], but specific ubiquitin ligase(s) for tyrosinase have not been identified [44]. We here demonstrate that tyrosinase (but not Tyrp-1) in B16 melanoma cells undergoes ubiquitination and subsequent degradation in lysosomes. While tyrosinase forms a heterodimer with Tyrp-1 [45], our observations detected ubiquitination solely on tyrosinase but not on Tyrp-1 (IP: anti-Ub in Figure 1A,B). This finding is consistent with the sensitivity of tyrosinase to LPIs compared with that of Tyrp-1 (the quantitative data of Figure 1A,B).

Based on the observation that the degree of tyrosinase ubiquitination is specifically altered by both inulavosin and PTU (Figure 1C), we hypothesize that inulavosin and PTU may exert differential effects on the ratio of ubiquitinated to non-ubiquitinated tyrosinase. Inulavosin is postulated to inhibit copper loading onto tyrosinase, whereas PTU functions by binding to tyrosinase as a pseudo substrate [28,30]. These distinct mechanisms potentially correlate with variations in the ubiquitinated to non-ubiquitinated tyrosinase ratio.

We hypothesized the existence of tyrosinase-specific ubiquitin ligase(s) in melanocytes that regulate tyrosinase turnover and cell pigmentation. By performing in vitro screening analysis, we identified a membrane-associated ubiquitin ligase termed RNF152, which specifically interacts with and ubiquitinates tyrosinase. In B16 cells, the expression levels of RNF152 were inversely correlated with those of tyrosinase and melanin (Figure 2). Given that the tyrosinase level directly influences melanin content, these findings strongly suggest that RNF152 plays a pivotal role in the regulation of melanogenesis in melanocytes.

The co-expression of RNF152-myc and tyrosinase-HA in HEK293T cells revealed a broad band (Figure 3A, lane 2), with increased signals in the presence of LPIs or BafA1 (Figure 3A, lanes 3 and 4, respectively). Because tyrosinase is known to undergo multiple post-translational modifications, it is plausible that the overexpression of RNF152-myc potentially disrupts tyrosinase’s modifications. Additionally, the tyrosinase profile expressed in HEK293T cells might differ from that of the endogenous tyrosinase in B16 cells. Indeed, a report has indicated differences in the size of tyrosinase in heterologous cellular systems like COS7 cells compared with wild-type mouse melanocytes, possibly due to incomplete complex carbohydrate processing [46]. These distinctions in profiles may result from the heterologous cellular systems used.

In cells lacking melanosomes, such as HeLa cells, exogenously expressed tyrosinase localizes in lysosomes [47,48,49]. RNF152 is primarily localized in LEs and lysosomes, where it undergoes self-ubiquitination, leading to its rapid degradation due to lysosomal processes [12,50]. Upon co-expression of RNF152-myc and tyrosinase-HA, we observed their predominant localization in the TGN. However, in the presence of lysosomal inhibitors, we found them in both LEs and lysosomes (Figure 3B,C).

It seems likely that RNF152 interacts with tyrosinase, and ubiquitinates both itself and tyrosinase in the TGN. Subsequently, these ubiquitinated molecules might be directed to intraluminal vesicles of MVBs and degraded by lysosomal proteases. Given that self-ubiquitinated RNF152 necessitates ESCRT (Endosomal Sorting Complex Required for Transport) for its transport to intraluminal vesicles of MVBs [50], exploring whether ubiquitinated tyrosinase utilizes this ESCRT-dependent pathway is warranted.

Both the RING and TM domains of RNF152 are essential for effective tyrosinase ubiquitination. The ubiquitination capability was lost in the RNF152(ΔTM) mutant but partially retained in the RNF152(C/S) mutant. This suggests that, besides the RING domain, RNF152 might harbor an additional binding site for E2 ubiquitin-conjugating enzyme(s), including Ubc13, an E2 enzyme required for RagA ubiquitination by RNF152 [13], despite the lack of documented evidence in the literature. Conversely, the ΔTM mutant lost its ubiquitination ability due to its inability to interact with tyrosinase. Previous studies have reported similar findings regarding RNF152 self-ubiquitination and its involvement in apoptosis. While self-ubiquitination and apoptotic activity are still detected in the C/S mutant, they are entirely absent in the ΔTM mutant [12,50]. Notably, the cytoplasmic localization of the ΔTM mutant likely parallels its reduced binding capacity to tyrosinase and diminished ubiquitination activity.

Hence, the functional role of RNF152 likely relies on subcellular localization and the TM-domain-dependent recognition of substrates. For instance, MARCH8, another RING-finger ubiquitin ligase, interacts with its substrates (e.g., TfR and HLA-DR) and ubiquitinates them in a TM-domain-dependent manner [9,51]. However, the consensus signal for TM-domain recognition by MARCH8 is yet to be identified. Further investigations are required to demonstrate the specificity of TM-domain-dependent recognition of tyrosinase by RNF152.

**Figure 7:**
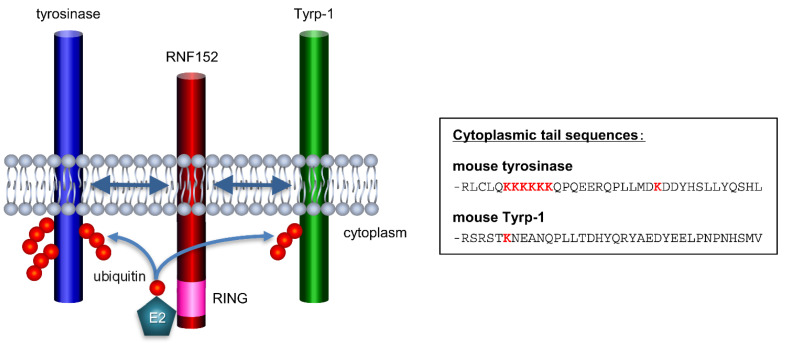
Putative mechanism of RNF152 interaction and ubiquitination of tyrosinase and Tyrp-1 (schematic). The TM domain of RNF152 interacts with the TM domains of tyrosinase and Tyrp-1. In addition, RNF152 interacts with an unknown E2 ubiquitin-conjugating enzyme, and the E2 conjugates ubiquitin to lysine residue(s) of CT domains of tyrosinase and Tyrp-1. Tyrosinase undergoes stronger ubiquitination than Tyrp-1, likely due to the cytoplasmic tail of mouse tyrosinase harboring seven lysine residues, whereas that of mouse Tyrp-1 has only one.

Normally, the ubiquitination of membrane proteins typically occurs at lysine residues located in the cytoplasmic region [7]. Based on this, Figure 7 illustrates schematic structures of tyrosinase, RNF152, and Tyrp-1 in a membrane model, along with a comparative amino acid sequence of the cytoplasmic tails of mouse tyrosinase versus mouse Tyrp-1 [49]. The cytoplasmic tail of mouse tyrosinase contains seven lysine residues, whereas that of mouse Tyrp-1 has only one. This distinction might explain why RNF152, despite its interaction with Tyrp-1, cannot efficiently ubiquitinate Tyrp-1.

In conclusion, RNF152 evidently regulates tyrosinase protein turnover primarily through its ubiquitination activity, thereby modulating cell pigmentation by controlling the expression level of tyrosinase, a pivotal enzyme in melanin synthesis among melanocytes. Thus, compounds or factors that augment the expression levels of ubiquitin ligase(s) targeting tyrosinase as substrate, similar to RNF152, hold potential as innovative whitening reagents [36].

## Figures and Tables

**Table 1:** E3 ubiquitin ligases with higher affinity for tyrosinase in the presence of inulavosin.

E3 Binding Assay Result to V5-Tyrosinase					Predicted Localization *	Predicted Number of Transmembrane Domains *
		Sample/Mock		Inulavosin/DMSO		
Symbol	Rank	DMSO	Inulavosin			
RNF152	1	16.65	22.13	1.33	Lysosome	1
VPS41	2	17.99	20.97	1.17	Cytosol, lysosome, Golgi, endosome	0
RNF41	3	17.84	20.55	1.15	Cytosol	0
ZNF598	4	16.43	18.52	1.13	Cytosol	0
TRIM21	5	19.82	21.73	1.10	Cytosol, nucleus	0

* Predictions were obtained from the following sites: GeneCard: “https://www.genecards.org (25 November 2023)”; Uniprote: “https://www.uniprot.org (25 November 2023)”

## Data Availability

The data presented in this study are available on request from the corresponding authors.

## Supplementary Materials

The following supporting information can be downloaded at: https://www.mdpi.com/article/10.3390/membranes14020043/s1, Figure S1: Amino acid sequence of RNF152 and evaluation of the antibody to RNF152. (A) In the amino acid sequence of mouse RNF152, cysteine residues mutated in C/S mutant are indicated in red font, whereas the transmembrane (TM) domain is highlighted in yellow. The peptide sequence used for rabbit immunization is highlighted in green. (B) An affinity purified antibody against synthetic peptide of RNF152 reacted with multiple bands on immunoblots of cell lysates from B16 melanoma and HEK293T cells. Upon the addition of an excess amount of synthetic peptide, only one band close to the predicted molecular weight of RNF152 (22.4 kDa) was reduced, suggesting that this diminished band corresponds to RNF152 (red arrow), while the others are nonspecific (red asterisks); Figure S2: HeLa cells transfected with RNF152-myc (wild-type [WT], C/S mutant, or ΔTM mutant) were subjected to immunofluorescence analyses using anti-myc and anti-syntaxin 6 (TGN marker) antibodies. Both WT and the C/S mutant exhibited localization to the TGN, whereas the ΔTM mutant was observed in the cytosol. Scale bar: 10 μm; Table S1: E3 ubiquitin ligases with higher affinity for tyrosinase in the presence of inulavosin.

## Abbreviations

ERAD: ER-associated degradation; IP, immunoprecipitation; LEs, late endosomes; LPIs, lysosomal protease inhibitors; MVB, multivesicular body; PTU, phenylthiourea; RING, Really Interesting New Gene; RNF, RING finger; TGN, trans-Golgi network; TM, transmembrane; and Tyrp-1, tyrosinase-related protein-1.
