# Supplementary material for: Membrane-Associated Ubiquitin Ligase RING Finger Protein 152 Orchestrates Melanogenesis via Tyrosinase Ubiquitination
Source: Membranes (Basel). 2024 Feb 1;14(2):43. doi: 10.3390/membranes14020043 (PMC10890620; doi:10.3390/membranes14020043)
Supplement: Supplementary file 1 [file membranes-14-00043-s001.zip › Supplementary Figures/Figs. S1 and S2.pdf]

Supplementary material

Membrane-associated ubiquitin ligase RNF152 orchestrates melanogenesis via tyrosinase ubiquitination

Ryota Ueda, Rina Hashimoto, Yuki Fujii, José C.J.M.D.S. Menezes, Hirotaka Takahashi,
Hiroyuki Takeda, Tatsuya Sawasaki, Tomonori Motokawa,
Kenzo Tokunaga, and Hideaki Fujita

A

```
1   metlsqdsll ecqicfnyys prrrpklldc khtccsvclq qmrtsqkdvr cpwcrgitkl
61  ppgfsvsqlp ddpevlavia iphtsehtpv fiklpsngcy mlplpisker tllpgdmgcr
121 llpgsqqksl tvvtipaeqq plqqgappea veeepdrrgv vksstwsgvc tvilvacvlv
181 fllgivlhnm sciskrftvi scg
```

B

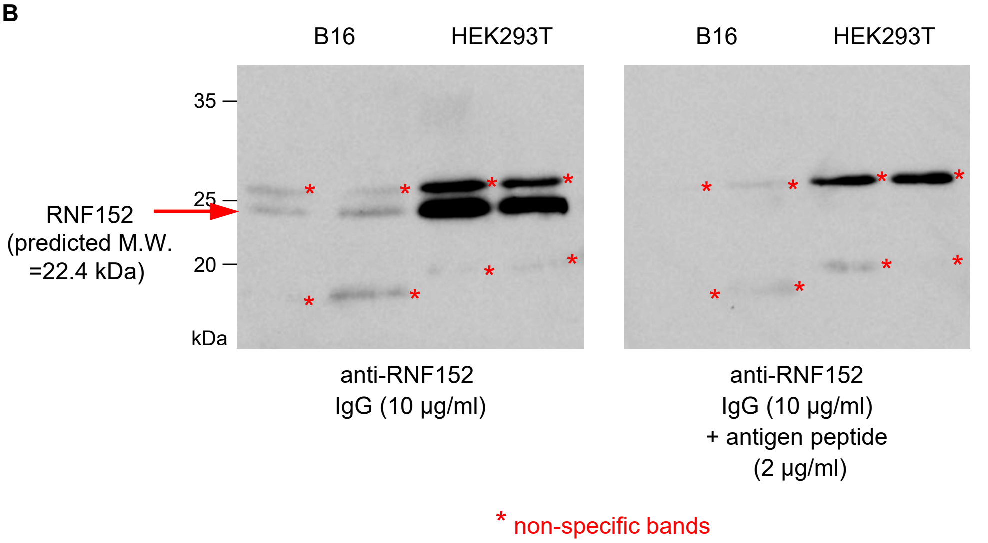

Figure S1. Amino acid sequence of RNF152 and evaluation of the antibody to RNF152. **(A)** In the amino acid sequence of mouse RNF152, cysteine residues mutated in C/S mutant are indicated in red font, whereas the transmembrane (TM) domain is highlighted in yellow. The peptide sequence used for rabbit immunization is highlighted in green. **(B)** An affinity purified antibody against synthetic peptide of RNF152 reacted with multiple bands on immunoblots of cell lysates from B16 melanoma and HEK293T cells. Upon the addition of an excess amount of synthetic peptide, only one band close to the predicted molecular weight of RNF152 (22.4 kDa) was reduced, suggesting that this diminished band corresponds to RNF152 (red arrow), while the others are non-specific (red asterisks).

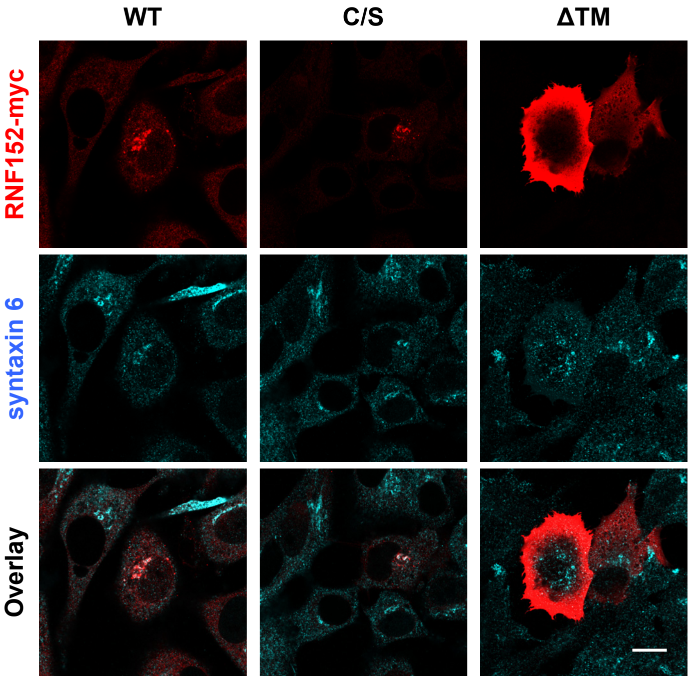

Figure S2. HeLa cells transfected with RNF152-myc (wild-type [WT], C/S mutant, or ΔTM mutant) were subjected to immunofluorescence analyses using anti-myc and anti-syntaxin 6 (TGN marker) antibodies. Both WT and the C/S mutant exhibited localization to the TGN, whereas the ΔTM mutant was observed in the cytosol. Scale bar: 10 µm.
